# Relationship between environment factors and the number of outpatient visits at a clinic for nonallergic rhinitis in Japan, extracted from electronic medical records

**DOI:** 10.1186/s40001-015-0151-3

**Published:** 2015-07-08

**Authors:** Takayuki Hoshino, Ayami Hoshino, Junya Nishino

**Affiliations:** Information Management Officer, Department of Clinical Research and Informatics, National Center for Global Health and Medicine, Tokyo, Japan; Akagi-kohgen Hospital, Gunma, Japan; Department of Internal Medicine, Gohyakuyama Clinic, Gunma, Japan; Graduate School of Environmental Information, Teikyo Heisei University, Tokyo, Japan

**Keywords:** Nonallergic rhinitis, Electronic medical records, Weather, Environment, Biometeorology

## Abstract

**Background:**

The objective of this study was to evaluate the influence of the environmental factors (meteorological factors, air pollutant levels, etc.) on the number of clinic consultations for nonallergic rhinitis (NAR).

**Methods:**

Among the 9056 outpatients visiting a general internal medicine clinic in Japan between August 2012 and the end of July 2013 (counting return visitors as multiple cases), the total daily number of first visits for NAR plus the number of extraordinary visits by patients with NAR for acute exacerbation of the disease was investigated using electronic medical records and analyzed.

**Results:**

Major parameters with significant Spearman’s correlation coefficients and significant correlation coefficients also in the multiple regression analysis were the mean vapor pressure (coefficient of determination 27.3 %) throughout the year, mean vapor pressure (58.4 %), mean temperature (44.4 %), maximum 10-min precipitation (12.0 %) only during the autumn-winter period, and temperature difference (13.3 %) only during the spring-summer period.

**Conclusions:**

The mean vapor pressure is the most important environmental factor associated with acute exacerbation of NAR.

## Background

Nonallergic rhinitis (NAR) is currently considered as a condition that must be diagnosed by exclusion [1–3]. A diagnosis of NAR requires negative specific IgE responses on skin or serologic testing [4, 5]. Symptoms are classically aggravated by irritant triggers such as tobacco smoke, perfumes/fragrances, and temperature or barometric pressure changes [6–12]. Symptoms and signs can closely resemble those of allergic rhinitis and can be difficult to differentiate from those of allergic rhinitis. While a careful history, physical examination, and diagnostic testing help clinicians to arrive at a definitive diagnosis, treatment can be challenging [11]. Treatment options for intractable rhinitis include antihistamines, topical or systemic corticosteroids, topical anticholinergics, laser or radiofrequency tissue volume reduction of turbinates, and subcutaneous or sublingual immunotherapy [12–17].

Some patients with intractable rhinitis complain of persistent and severe nasal symptoms in spite of prolonged treatment [17]. It is therefore useful to identify environmental factors that can aggravate the symptoms of rhinitis in NAR patients and to avoid exposure to such aggravating factors during daily living. With the recent spread of implements such as air conditioners, humidifiers, humidity removers, and air cleaners in households and the increasing social attention to environmental adjustment, a more accurate identification of biometeorological aggravating factors may help in improving the quality of life (QOL) of NAR patients.

Under the universal health insurance system in Japan, patients in Japan can visit medical facilities at their own discretion depending on the severity of subjective symptoms (a situation called “free access”). Particularly in urban areas, like the community in which the Gohyakuyama Clinic (the site for this study) is located, at least one medical facility is available within easy reach (within several km) for each patient. Under such circumstances, individual patients can easily receive medical care upon aggravation of symptoms on their day off. Furthermore, a public monitoring station in the close vicinity of the clinic (about 15 km from the clinic, consistent with the radius of the medical care zone around this clinic) publishes detailed data needed for the analysis.

A number of studies have been conducted to identify factors aggravating NAR, but analyses of environmental factors primarily relied on information collected from patient questionnaire surveys [9, 12, 18, 19]. Also, there have been fewer reports on the relationship between post-aggravation hospital/clinic visits and environmental factors as compared to similar reports for the case of allergic rhinitis or asthma [14, 20–23].

The present study was undertaken to analyze the association between the number of clinic consultations for NAR and various environmental factors using the 1-year accumulated data on patient visits to the clinic collected from electronic medical records, after obtaining informed consent from individual patients.

## Methods

### Patients

Outpatients with clinical presentation of NAR were detected from among patients presenting to the General Medicine Department of Gohyakuyama Clinic, between August 2012 and July 2013. Patients were qualified for inclusion in this study if they fulfilled the diagnosis criteria of NAR listed in Table 1. Not only patients making an initial visit for NAR, but also patients making extraordinary visits for acute exacerbation of chronic symptoms were included in the study.

**Table 1:** Diagnosis criteria of NAR

Category	Criteria
A	Perennial symptoms of rhinitis are present
B	Three major symptoms (sneezing, nasal discharge, and nasal congestion) are induced by environmental changes, at some time of the day or by stress
C	Pathologic allergens responsible for perennial subjective symptoms were absent (or unknown) in the blood tests carried out to check for IgE specific to representative allergens of inhalational or dietary origin conducted at any otorhinolaryngology clinic or our clinic

Information was extracted from the medical records of outpatient clinic attendees and correlated with the environmental data. Patients fulfilling all of the criteria shown were defined as having nonallegic rhinitis

The study protocol was pre-approved by the “Clinical Research and Ethics Committee of Aoki Hospital”, Hakuseikai Healthcare Corporation, Honjyou City, Saitama, Japan. Oral informed consent was obtained from each of the participating patients in this study.

### Analytical processes and statistical analyses

We had the successful experience of analyzing relationship between environment factors and the number of outpatient visits for hypotension by the same method [24].

#### Data from nonallergic rhinitis patients

From the clinical data recorded in the electronic medical records during the 1-year period of this study, those on the first visits by NAR patients and on extraordinary visits by patients with acute exacerbation of the chronic symptoms of NAR were extracted for the analysis. Periodic visits made in response to the physician’s instructions were excluded from the analysis. Repeated visits by the same patient for aggravation of NAR symptoms were counted as separate records. Subsequently, the date of the first visit for NAR and the patient count of first visits were extracted from all attendee records. Then, excluding the repeated visits made by these patients in response to the physician’s instruction, extraordinary visits for aggravation of NAR were counted and combined with the patient count of first visits to yield the total number of clinic consultations for NAR on each day of the study period.

The outpatient universe of clinic in this study could be liberally divided into three groups: individual proprietor who traditionally had a day off on Wednesdays; retired people and farmers who have freedom over their time; and an employed person who has days off on Saturdays and Sundays.

Gohyakuyama Clinic is closed every Thursday and Sunday. The 5 days on which the Gohyakuyama Clinic was open included at least 1 day off for each patient category, enabling the entire patient population to visit the clinic over a given week. Thus, the 1-week moving average (i.e., the current day and anterior 6 days) was calculated. This 1-week moving average was adopted for the analysis with the aim of bias elimination of the number of NAR outpatients.

#### Environment factors

Because the majority (89 %) of outpatients came from within a radius of 20 km from the clinic, environmental data for the period from August 2012 to July 2013 published for Maebashi City, Gunma Prefecture, by the Maebashi Weather Station and Gunma Prefectural Institute of Public Health and Environmental Sciences, which is only 15 km away from the clinic, were evaluated. The 1-week moving averages of the environmental data were directly used for the statistical analyses. This 1-week moving average was used for the analysis with the aim of bias elimination of the time lapse between the environmental event and the day of the patient visit for NAR symptoms. Spearman’s correlation coefficient was calculated for the 1-week moving average of the number of clinic consultations for NAR and the 1-week moving averages of the environmental data.

The environmental data recorded during this study period included the following: mean and minimum sea-level pressures; mean station pressure; maximum hourly precipitation; maximum 10-min precipitation; total precipitation; mean, maximum, and minimum temperatures; temperature difference (difference between the minimum temperature and maximum temperature in a day); mean and minimum humidities; mean vapor pressure; mean and maximum wind speeds; maximum instantaneous wind speed; total global solar radiation; duration of daylight; maximum snow depth; total snowfall; mean cloud cover; and atmospheric levels of carbon monoxide (CO), sulfur dioxide (SO_2_), nitric oxide (NO), nitric oxide (NOX), nitrogen dioxide (NO_2_), photochemical oxidant (OX), non-methane hydrocarbons (NMHC) and methane (CH_4_), total atmospheric hydrocarbon content (THC), and suspended particulate matter (SPM).

### Statistical analyses

All statistical analyses were performed with SPSS® version 11.0 (SPSS Inc. Chicago, IL, USA) for Windows® and Spearman’s correlation coefficient after Bonferroni’s correction. Statistical significance was assumed at a two-tailed *p* value of <0.05.

After the 1-week moving average for all the NAR patients and the Spearman’s coefficient of correlation with the environmental factors were calculated, the partial correlation coefficient among similar parameters was calculated. When the analyses revealed very strong correlations (*r* ≥ 0.9) for two parameters, suggesting that the changes in the two parameters were evidently related to the same factors, we rearranged the presentation of the parameters to be analyzed. For example, maximum hourly precipitation which is larger in terms of the *p* value of the coefficient of correlation with the number of NAR patients than the maximum 10-min precipitation was excluded. The variables excluded are listed in Table 2. Then, forced-entry multiple regression analysis was carried out for all the NAR patients with the 1-week moving average serving as the dependent variable and the environmental factors serving as independent variables. From the standard regression coefficient (*β*) and correlation coefficient (*r*) obtained thus, the coefficient of determination (*β* × *r*) was determined. Variables with significant Spearman’s correlation coefficients and significant regression coefficients also in the multiple regression analysis are marked with an asterisk in Table 2.

**Table 2:** Relationship with environmental factors in the entire population of NAR patients

Meteorological event	Mean	SD	Spearman’s correlation coefficient (*r*)		*p*	Number	Exclusion^a^	Multiple regression analysis correlation coefficient^b^		SE	Standard regression coefficient (*β*)	*t*	*p*	Coefficient of determination^d^ (*β* × *r*)	
Mean station pressure (hPa)	997.250	19.490	−0.013		0.800	365		0.006		0.003	0.094	1.856	0.064	<0.001	
Mean sea-level pressure (hPa)	1010.760	19.719	0.019		0.720	365	+								
Minimum sea-level pressure (hPa)	1009.760	3.850	−0.107	*	0.041	365	+								
Total precipitation (mm)	2.080	3.064	0.087		0.098	365		0.025		0.035	0.058	0.724	0.470	0.005	
Maximum hourly precipitation (mm)	0.941	1.332	0.043		0.409	365	+								
Maximum 10-min precipitation (mm)	0.402	0.595	0.036		0.493	365		0.282		0.197	0.125	1.433	0.153	<0.001	
Mean temperature (°C)	15.321	9.009	−0.269	**	<0.0001	365		−0.146		0.300	−0.961	−0.488	0.626	0.259	
Maximum temperature (°C)	20.726	9.087	−0.252	**	<0.0001	365		0.208		0.317	1.372	0.655	0.513	−0.346	
Minimum temperature (°C)	10.888	9.161	−0.274	**	<0.0001	365	+								
Temperature difference (°C)^c^	9.838	1.639	0.205	**	<0.0001	365		0.281		0.221	0.344	1.274	0.204	0.071	
Mean vapor pressure (hPa)	12.354	8.064	−0.290	**	<0.0001	365		−0.162	**	0.055	−0.942	−2.942	0.003	0.273	*
Mean humidity (%)	58.978	10.226	−0.320	**	<0.0001	365		−0.042		0.030	−0.315	−1.373	0.171	0.101	
Minimum humidity (%)	38.117	9.961	−0.311	**	<0.0001	365		0.001		0.037	0.007	0.026	0.979	<0.001	
Mean wind speed (m/s)	3.019	0.638	0.330	**	<0.0001	365		−0.134		0.285	−0.063	−0.471	0.638	−0.021	
Maximum wind speed (m/s)	6.159	1.141	0.361	**	<0.0001	365		0.022		0.154	0.019	0.142	0.887	0.007	
Maximum instantaneous wind speed (m/s)	10.898	2.298	0.359	**	<0.0001	365	+								
Duration of daylight (h)	6.666	2.029	0.131	*	0.012	365		−0.165		0.129	−0.249	−1.280	0.202	−0.033	
Total global solar radiation (MJ/mm^2^)	15.098	4.701	0.148	**	0.005	365		−0.045		0.064	−0.156	−0.700	0.485	−0.023	
Total snowfall (cm)	0.017	0.087	0.065		0.216	365		−0.492		0.785	−0.032	−0.627	0.531	<0.001	
Maximum snow depth (cm)	0.022	0.122	0.065		0.214	365	+								
Mean cloud cover (%)	5.970	2.094	−0.148	**	0.005	365		0.326	**	0.090	0.505	3.606	<0.0001	−0.075	*
Sulfur dioxide (ppm)	0.002	0.000	−0.359	**	<0.0001	365		−551.346	**	193.043	−0.162	−2.856	0.005	0.058	*
Nitric oxide (ppm)	0.001	0.001	−0.409	**	<0.0001	365		−251.464	**	95.798	−0.220	−2.625	0.009	0.090	*
Nitrogen dioxide (ppm)	0.008	0.002	−0.147	**	0.005	365		−111.394		66.856	−0.153	−1.666	0.097	0.022	
Nitrogen oxides (ppm)	0.010	0.003	−0.212	**	<0.0001	365	+								
Carbon monoxide (ppm)	0.228	0.056	0.145	**	0.006	365		4.641		3.277	0.193	1.416	0.158	0.028	
Photochemical oxidant (ppm)	0.034	0.009	0.404	**	<0.0001	365		−10.868		27.290	−0.070	−0.398	0.691	−0.028	
Non-methane hydrocarbons (ppbC)	0.203	0.069	−0.165	**	0.002	365		1.951		1.541	0.100	1.266	0.206	−0.017	
Methane (ppbC)	1.958	0.059	−0.400	**	<0.0001	365		−2.079		1.214	−0.082	−1.713	0.088	0.033	
Total hydrocarbon content (ppbC)	2.161	0.101	−0.208	**	<0.0001	365	+								
Suspended particulate matter (mg/m^3^)	0.015	0.007	0.017		0.748	365		31.455		20.917	0.151	1.504	0.134	<0.001	

*<0.05; **<0.01

^a^Factors with partial correlations of >0.9 were excluded from one side (+)

^b^Forced-entry multiple regression analysis with the 1-week moving average of the total number of NAR patients serving as the dependent variable and environmental factors serving as independent variables (*F* = 17.327, *p* < 0.001, *R* ^2^ = 0.546)

^c^Maximum temperature – minimum temperature

^d^Coefficients of determination calculated from significant correlation coefficients and significant regression coefficients are marked with an asterisk

Because the moving average of the total number of NAR patients showed large seasonal variations as shown in the “Results” section, we conducted a secondary analysis of the data to examine whether or not major environmental factors affecting the number of NAR patients also differed significantly among the seasons by dividing the year into the spring-summer period and the autumn-winter period. Because meteorological variables with partial correlation coefficients of ≥0.9 were considered to change on the basis of the same factors, overlapping parameters (those marked with a plus sign in Tables 3 and 4) were excluded, and the remaining parameters were included in the multiple regression analysis.

**Table 3:** Relationship with environmental factors in NAR patients visiting the clinic only during the autumn-winter period

Meteorological event	Mean	SD	Spearman’s correlation coefficient (*r*)		*p*	Number	Exclusion^a^	Multiple regression analysis Correlation coefficient^b^		SE	Standard regression coefficient (*β*)	*t*	*p*	Coefficient of determination^d^ (*β* × *r*)	
Mean station pressure (hPa)	995.762	27.124	0.056		0.449	184		−0.001		0.002	−0.032	−0.470	0.638	−0.002	
Mean sea-level pressure (hPa)	1009.260	27.401	−0.017		0.821	184	+							<0.0001	
Minimum sea-level pressure (hPa)	1011.090	2.929	0.044		0.555	184		0.008		0.010	0.058	0.832	0.406	0.003	
Total precipitation (mm)	2.148	3.265	0.475	**	<0.0001	184		0.011		0.018	0.063	0.628	0.530	0.030	
Maximum hourly precipitation (mm)	1.065	1.506	0.485	**	<0.0001	184	+							<0.0001	
Maximum 10-min precipitation (mm)	0.436	0.640	0.513	**	<0.0001	184		0.215	*	0.099	0.233	2.168	0.031	0.120	*
Mean temperature (°C)	15.222	9.917	0.375	**	<0.0001	184		0.074	**	0.023	1.183	3.164	0.002	0.444	*
Maximum temperature (°C)	20.440	10.094	0.382	**	<0.0001	184	+							<0.0001	
Minimum temperature (°C)	10.973	9.914	0.369	**	<0.0001	184	+							<0.0001	
Temperature difference (°C)^c^	9.468	0.931	−0.043		0.562	184		0.176	**	0.043	0.526	4.113	<0.0001	−0.023	*
Mean vapor pressure (hPa)	12.957	8.644	0.382	**	<0.0001	184		−0.107	**	0.025	−1.526	−4.369	<0.0001	−0.584	*
Mean humidity (%)	61.053	8.040	0.557	**	<0.0001	184		0.008		0.014	0.143	0.538	0.591	0.080	
Minimum humidity (%)	39.784	7.539	0.518	**	<0.0001	184	+							<0.0001	
Mean wind speed (m/s)	2.874	0.455	−0.442	**	<0.0001	184		0.184		0.143	0.211	1.282	0.201	−0.093	
Maximum wind speed (m/s)	5.880	0.840	−0.319	**	<0.0001	184		−0.118		0.074	−0.242	−1.596	0.111	0.077	
Maximum instantaneous wind speed (m/s)	10.395	1.646	−0.358	**	<0.0001	184	+							<0.0001	
Duration of daylight (h)	6.669	1.554	−0.322	**	<0.0001	184		−0.030		0.059	−0.110	−0.507	0.612	0.035	
Total global solar radiation (MJ/mm^2^)	13.078	4.397	0.268	**	0.000	184		−0.036		0.033	−0.306	−1.095	0.274	−0.082	
Total snowfall (cm)	0.000	0.000	-		-	184	+							<0.0001	
Maximum snow depth (cm)	0.000	0.000	-		-	184	+							<0.0001	
Mean cloud cover (%)	5.593	1.915	0.317	**	<0.0001	184		0.086	*	0.044	0.327	1.986	0.048	0.104	*
Sulfur dioxide (ppm)	0.002	0.000	−0.665	**	<0.0001	184		11.699		97.038	0.008	0.121	0.904	−0.005	
Nitric oxide (ppm)	0.002	0.001	−0.046		0.537	184		−119.889	*	48.946	−0.256	−2.449	0.015	0.012	*
Nitrogen dioxide (ppm)	0.009	0.002	−0.409	**	<0.0001	184		−49.753		34.335	−0.167	−1.449	0.148	0.068	
Nitrogen oxides (ppm)	0.010	0.003	−0.302	**	<0.0001	184	+							<0.0001	
Carbon monoxide (ppm)	0.231	0.054	−0.418	**	<0.0001	184		2.780	*	1.585	0.282	1.754	0.080	−0.118	*
Photochemical oxidant (ppm)	0.028	0.005	0.038		0.606	184		−18.443		14.386	−0.288	−1.282	0.201	−0.011	
Non-methane hydrocarbons (ppbC)	0.181	0.084	−0.157	**	0.033	184		0.095		0.814	0.012	0.117	0.907	−0.002	
Methane (ppbC)	1.956	0.030	−0.035		0.634	184		0.150		0.615	0.014	0.244	0.807	0.000	
Total hydrocarbon content (ppbC)	2.137	0.101	−0.116		0.118	184	+							<0.0001	
Suspended particulate matter (mg/m^3^)	0.012	0.005	0.224	**	0.002	184		−3.327		10.619	−0.039	−0.313	0.754	−0.009	

*<0.05; **<0.01

^a^Factors with partial correlations of >0.9 were excluded from one side (+)

^b^Forced-entry multiple regression analysis with the 1-week moving average of number of NAR patients visiting the clinic only during the autumn season for aggravation of symptoms as a dependent variable and environmental factors serving as independent variables (*F* = 28.136, *p* < 0.001, *R* ^2^ = 0.785)

^c^Maximum temperature – minimum temperature

^d^Coefficients of determination calculated from significant correlation coefficients and significant regression coefficients are marked with an asterisk

**Table 4:** Relationships with environmental factors in NAR patients visiting the clinic during only the spring-summer period

Meteorological event	Mean	SD	Spearman’s correlation coefficient (*r*)		*p*	Number	Exclusion^a^	Multiple regression analysis Correlation coefficient^b^		SE	Standard regression coefficient (*β*)	*t*	*p*	Coefficient of determination^d^ (*β* × *r*)	
Mean station pressure (hPa)	998.764	3.955	0.266	**	0.000	165		−0.119		0.066	−0.421	−1.812	0.072	−0.112	
Mean sea-level pressure (hPa)	1012.290	4.285	0.318	**	<0.0001	165	+								
Minimum sea-level pressure (hPa)	1008.410	4.199	0.098		0.189	165		0.056		0.060	0.214	0.928	0.355	0.021	
Total precipitation (mm)	2.012	2.852	−0.205	**	0.006	165		0.007		0.027	0.020	0.263	0.793	−0.004	
Maximum hourly precipitation (mm)	0.815	1.117	−0.269	**	0.000	165	+								
Maximum 10-min precipitation (mm)	0.368	0.546	−0.267	**	0.000	165		−0.319		0.206	−0.172	−1.545	0.125	0.046	
Mean temperature (°C)	15.421	8.007	−0.596	**	<0.0001	165		−0.061		0.057	−0.437	−1.074	0.285	0.260	
Maximum temperature (°C)	21.017	7.952	−0.570	**	<0.0001	165	+								
Minimum temperature (°C)	10.802	8.354	−0.582	**	<0.0001	165	+								
Temperature difference (°C)^c^	10.215	2.066	0.394	**	<0.0001	165		0.164	**	0.061	0.338	2.679	0.008	0.133	*
Mean vapor pressure (hPa)	11.741	7.402	−0.548	**	<0.0001	165		−0.079		0.060	−0.517	−1.318	0.190	0.283	
Mean humidity (%)	56.868	11.699	−0.538	**	<0.0001	165		−0.026		0.028	−0.298	−0.936	0.351	0.160	
Minimum humidity (%)	36.423	11.711	−0.514	**	<0.0001	165	+								
Mean wind speed (m/s)	3.166	0.755	0.441	**	<0.0001	165		−0.521		0.281	−0.379	−1.852	0.066	−0.167	
Maximum wind speed (m/s)	6.442	1.325	0.527	**	<0.0001	165		0.001		0.120	0.002	0.012	0.990	0.001	
Maximum instantaneous wind speed (m/s)	11.409	2.720	0.508	**	<0.0001	165	+								
Duration of daylight (h)	6.662	2.422	0.358	**	<0.0001	165		−0.018		0.145	−0.044	−0.126	0.900	−0.016	
Total global solar radiation (MJ/mm^2^)	17.151	4.079	0.069		0.355	165		−0.187	*	0.080	−0.739	−2.332	0.021	−0.051	
Total snowfall (cm)	0.033	0.122	−0.152	**	0.042	165		−2.755	**	0.753	−0.315	−3.657	<0.0001	0.048	*
Maximum snow depth (cm)	0.044	0.171	−0.151	**	0.043	165	+								
Mean cloud cover (%)	6.354	2.201	−0.438	**	<0.0001	165		0.036		0.082	0.076	0.438	0.662	−0.033	
Sulfur dioxide (ppm)	0.002	0.001	−0.517	**	<0.0001	165		−367.808		187.422	−0.145	−1.962	0.052	0.075	
Nitric oxide (ppm)	0.001	0.001	−0.473	**	<0.0001	165		−78.669		462.055	−0.024	−0.170	0.865	0.011	
Nitrogen dioxide (ppm)	0.008	0.002	−0.226	**	0.002	165		−89.809		80.579	−0.136	−1.115	0.267	0.031	
Nitrogen oxides (ppm)	0.009	0.002	−0.321	**	<0.0001	165	+								
Carbon monoxide (ppm)	0.225	0.059	0.372	**	<0.0001	165		1.731		2.204	0.093	0.785	0.434	0.035	
Photochemical oxidant (ppm)	0.041	0.006	0.369	**	<0.0001	165		40.791		28.410	0.239	1.436	0.153	0.088	
Non - methane hydrocarbons (ppbC)	0.225	0.038	−0.377	**	<0.0001	165		5.745	**	1.543	0.209	3.722	<0.0001	−0.079	*
Methane (ppbC)	1.961	0.078	−0.456	**	<0.0001	165		−0.684		0.807	−0.047	−0.848	0.398	0.021	
Total hydrocarbon content (ppbC)	2.186	0.096	−0.536	**	<0.0001	165	+								
Suspended particulate matter (mg/m^3^)	0.018	0.007	−0.215	**	0.004	165		45.817	**	14.548	0.300	3.149	0.002	−0.064	*

*<0.05; **<0.01

^a^Factors with partial correlations of >0.9 were excluded from one side (+)

^b^Forced-entry multiple regression analysis with the 1-week moving average of the number of NAR patients visiting the clinic only during the spring-summer period for aggravation of symptoms as a dependent variable and environmental factors serving as independent variables (*F* = 33.053, *p* < 0.001, *R* ^2^ = 0.836)

^c^Maximum temperature – minimum temperature

^d^Coefficients of determination calculated from significant correlation coefficients and significant regression coefficients are marked with an asterisk

#### Secondary analysis on seasonal elements

##### Secondary analysis of NAR patients visiting the clinic for symptom aggravation only during the autumn-winter period

Six months from August 1, 2012 to January 31, 2013. NAR patients who visited the clinic for aggravation of symptoms only during the autumn-winter period were extracted. Patients who visited the clinic during the remaining half of the year (the spring-summer period) also were excluded. Like in the analysis of the entire population of NAR patients, the correlations of the number of clinical consultations for NAR aggravation with the environmental factors were explored in this population of autumn-winter visitors. After overlapping parameters (judged on the basis of the partial correlation coefficients) were excluded, multiple regression analysis was carried out to calculate the correlation of each environmental factor with the number of clinical consultations for NAR. Details of the analysis and the results are shown in Table 3.

##### Secondary analysis of NAR patients visiting the clinic for aggravation only during the spring-summer period

Six months from February 1, 2013 to July 31, 2013. NAR patients who visited the clinic for aggravation of symptoms only during the spring-summer period were extracted. Patients who also visited the clinic during the remaining half of the year (the autumn-winter period) were excluded. Like in the analysis of the entire population of NAR patients, the correlations of the number of clinical consultations for NAR aggravation with the environmental factors were explored in this population of spring-summer visitors. After overlapping parameters (judged on the basis of the partial correlation coefficients) were excluded, multiple regression analysis was carried out to calculate the correlation of each environmental factor with the number of clinical consultations for NAR. Details of the analysis and the results are shown in Table 4.

## Results

### Patients

In total, 9056 outpatients visited the General Internal Medicine Outpatient Department of Gohyakuyama Clinic between August 2012 and July 2013. Among these patients, there were 531 records of visits (including repeated visits) for the symptoms of NAR, with a gender (male/female) ratio of 112/419 and mean age of the patients of 48.2 ± 18.2 years (data are presented as the mean ± SD or number of patients).

### Correlations between the environmental data and consultations for nonallergic rhinitis

**Fig. 1:**
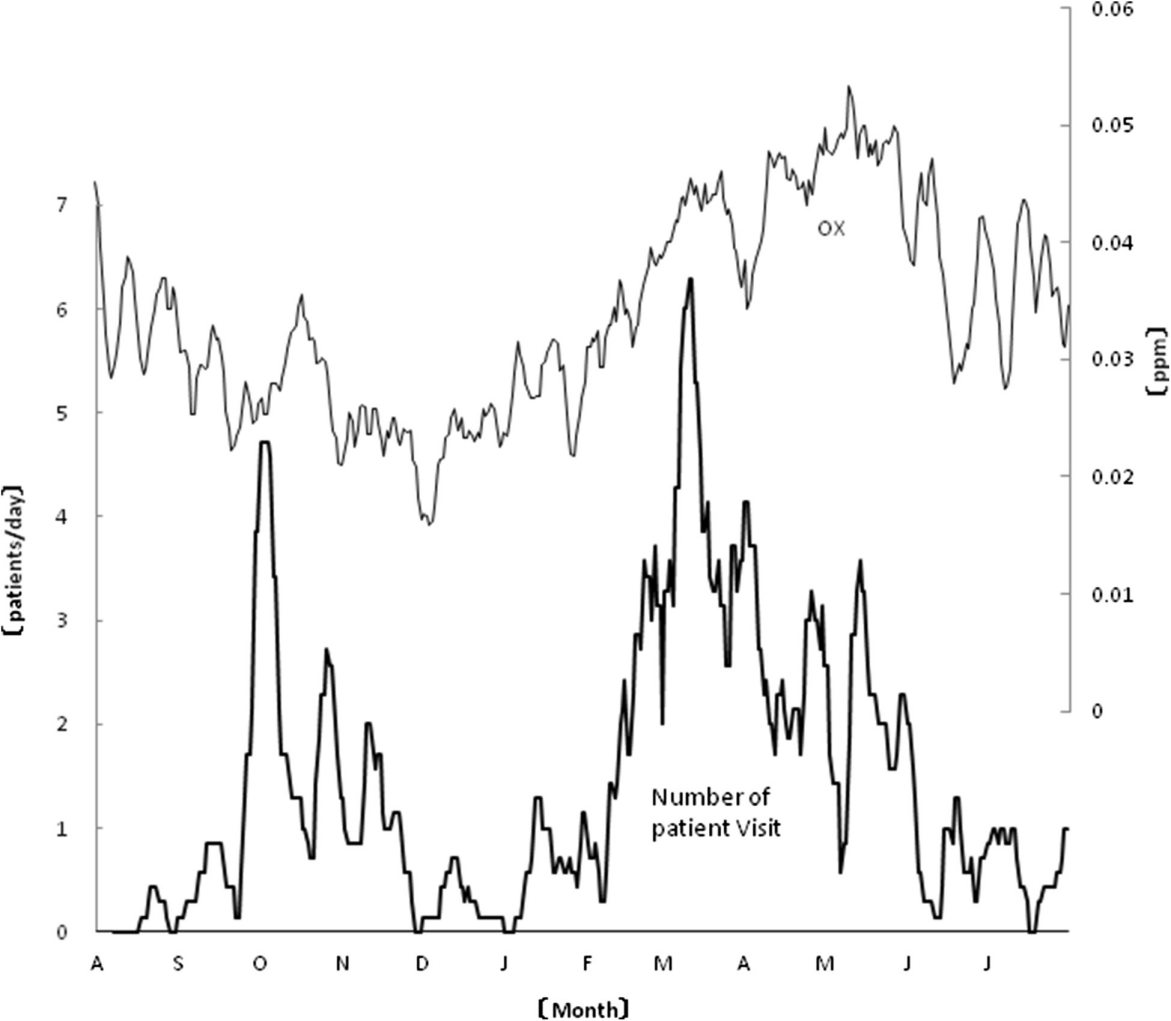
A significant correlation (0.404, *p* < 0.0001) was identified between the ambient air photochemical oxidant (OX) levels and the 7-day moving average of the number of clinic consultations for NAR in the studied Japanese cohort between August 2012 and July 2013

Environmental data for the study period are shown in Table 2. When 1-week moving averages of these data and of visits for NAR were analyzed, the strongest negative correlation (−0.409, *p* < 0.0001) was noted between NO and the 1-week moving average of the number of clinical consultations for NAR. The mean humidity (%), minimum humidity (%), and mean vapor pressure (hPa) also showed negative correlations with the number of clinical consultations for NAR. The minimum temperature (°C) and other temperature-related parameters also were negatively correlated with the number of clinical consultations for NAR. The highest positive correlation (0.404, *p* < 0.0001) was observed between the atmospheric OX levels and the 1-week moving average of the number of clinical consultations for NAR (Fig. 1). Conversely, a weak negative correlation between the daily total number of outpatient visits and the atmospheric OX level was observed (Spearman’s correlation coefficient, −0.219; *p* < 0.0001). Thus, the positive correlation with the OX level was unique to patients visiting for symptoms of NAR.

**Fig. 2:**
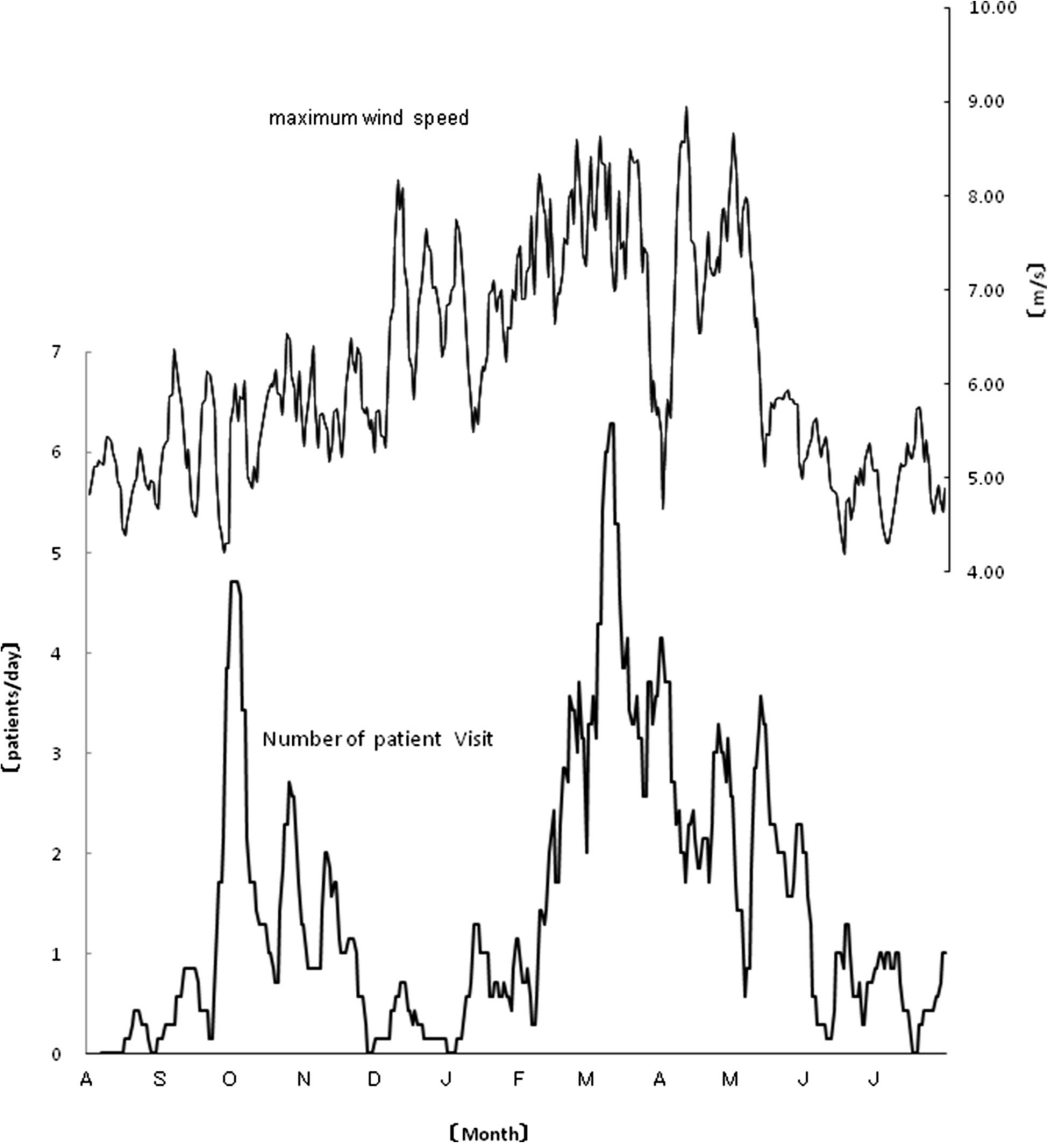
A significant correlation (0.361, *p* < 0.0001) was identified between the maximum wind speed and the 7-day moving average of the number of clinic consultations for NAR in the studied Japanese cohort between August 2012 and July 2013

**Fig. 3:**
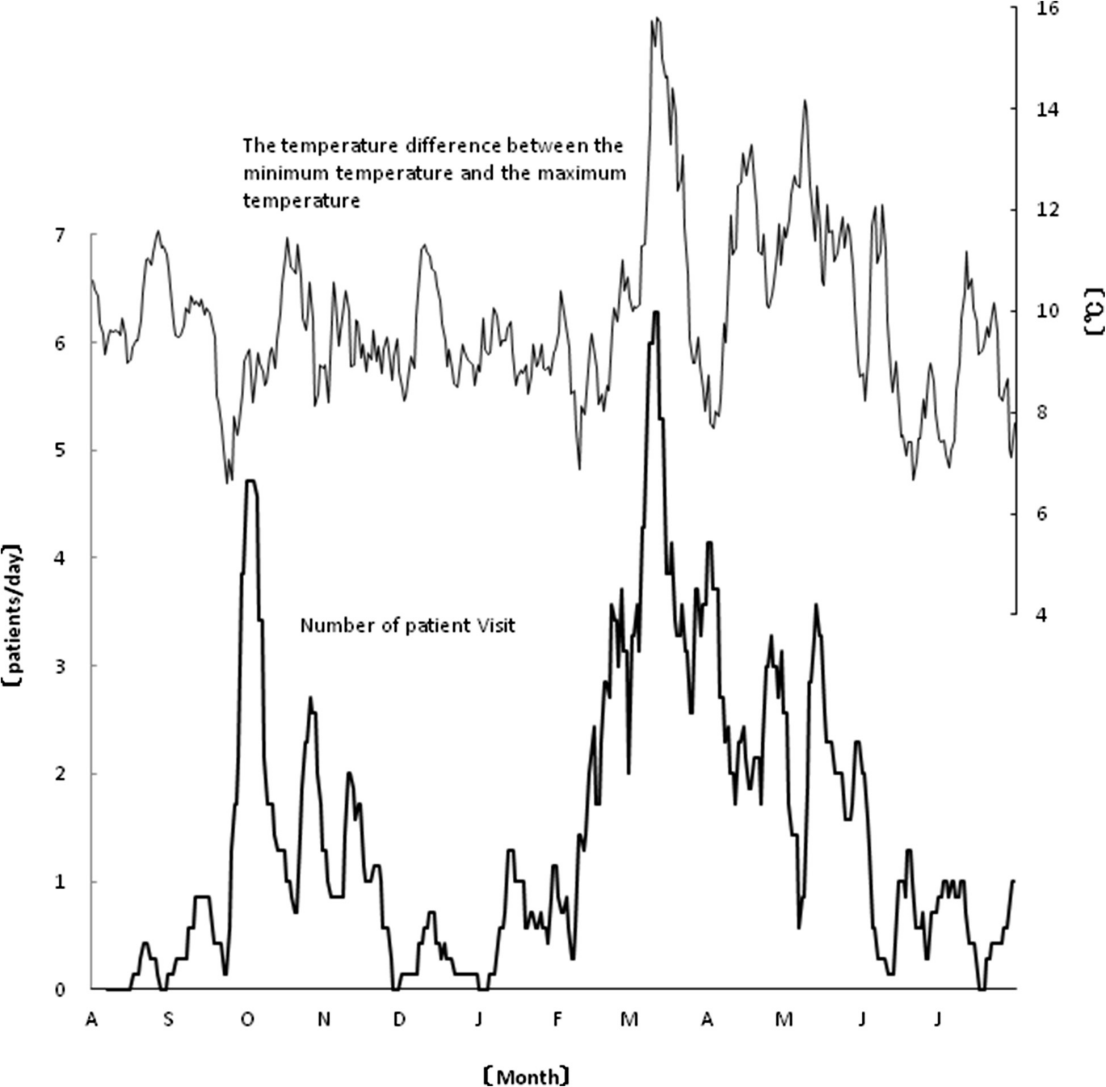
A significant correlation (0.205, *p* < 0.0001) was identified between the temperature difference (temperature difference between the minimum temperature and maximum temperature during the day) and the 7-day moving average of the number of clinic consultations for NAR in the studied Japanese cohort between August 2012 and July 2013

Other environmental factors that were significantly correlated with the 1-week moving average of the clinical consultation number for NAR were the wind speed (e.g., maximum wind speed, 0.361, *p* < 0.0001, Fig. 2) and temperature difference (0.205, *p* < 0.0001, Fig. 3). The data on the other factors are shown in Table 2, together with the statistical analysis methods. Factors for which significant Spearman’s correlation coefficients and significant regression coefficients also in the multiple regression analysis (*F* = 17.327, *p* < 0.001, *R*^2^ = 0.546) were obtained were the mean vapor pressure (coefficient of determination 27.3 %), mean cloud cover (7.5 %), atmospheric levels of sulfur dioxide (5.7 %), and atmospheric levels of nitric oxide (9.0 %).

### Results of secondary analysis on seasonal elements

#### Relationships between clinic visits for aggravation of NAR only during the autumn-winter period and environmental factors

The NAR patients visiting the clinic for aggravation only during the autumn-winter period accounted for 8.9 % (47 records) of all the NAR patients visiting the clinic for aggravation of symptoms (264 patients, 531 records). Forced-entry multiple regression analysis (*F* = 28.136, *p* < 0.001, *R*^2^ = 0.785) was carried out, with the 1-week moving average of the number of clinical consultations for NAR aggravation during the autumn-winter period serving as the dependent variable and the environmental factors serving as independent variables. In this analysis, major parameters with significant Spearman’s correlation coefficients and significant correlation coefficients also in the multiple regression analysis (*F* = 28.136, *p* < 0.001, *R*^2^ = 0.785) were the mean vapor pressure (coefficient of determination 58.4 %), mean temperature (44.4 %), and maximum 10-min precipitation (12.0 %).

#### Relationships between clinic visits for aggravation of NAR only during the spring-summer period and environmental factors

The NAR patients visiting the clinic for aggravation only during the spring-summer period accounted for 47.3 % (119 patients, 251 records) of all the NAR patients visiting the clinic for aggravation of symptoms (264 patients, 531 records). Forced-entry multiple regression analysis (*F* = 33.053, *p* < 0.001, *R*^2^ = 0.836) was carried out, with the 1-week moving average of the number of clinical consultations for NAR aggravation during the spring-summer period serving as the dependent variable and the environmental factors serving as independent variables. In this analysis, major parameters with significant Spearman’s correlation coefficients and significant correlation coefficients also in the multiple regression analysis (*F* = 33.053, *p* < 0.001, *R*^2^ = 0.836) were the temperature difference (coefficient of determination 13.3 %), non-methane hydrocarbons (7.9 %), suspended particulate matter (6.4 %), and total snowfall (4.8 %).

## Discussion

Among the meteorological factors, all the parameters related to wind speed (maximum wind speed, maximum instantaneous wind speed, and mean wind speed) showed a significant positive correlation with the number of clinic consultations for NAR in the present study. Classically, it has been reported that NAR patients tend to perceive sudden changes in temperature, relative humidity, or atmosphere pressure as factors aggravating their condition [6]. In the present study, the mean humidity (%), minimum humidity (%), and mean vapor pressure (hPa) showed slight negative correlations with the number of clinical consultations for NAR. If these relationships were combined with the empirical knowledge that the humidity level also affects the number of clinical consultations for NAR and that the humidity level negatively correlates with the number of clinical consultations for NAR, it may be reasonable to assume that dry weather with low humidity adversely affects NAR patients. In past studies of patients with asthma or allergic rhinitis who, like patients with NAR, are also known to be affected by autonomic imbalance, coldness has been pointed out as a major aggravating factor [25–27]. In the present study, three parameters related to the temperature, namely, the mean temperature (°C), maximum temperature (°C), and minimum temperature (°C), were also found to show slight negative correlations with the number of clinical consultations for NAR, which led us to confirm that exposure to cold weather can serve as a factor aggravating NAR also in NAR patients, just like in asthma patients for whom such a relationship has been empirically known. “Wind” which was shown to be correlated with the number of clinic consultations for NAR in the present study blows when there is a gradient in the temperature as well as atmosphere pressure, and passage of a cold front or warm front is accompanied by a change in the relative humidity. For this reason, under the weather condition characterized by strong wind, changes also occur in the temperature, atmosphere pressure, and relative humidity over time, and these sudden changes in the weather can serve as aggravating factors for NAR through their influence on the autonomic nervous system.

In regard to the temperature, data were available on both maximum temperature and minimum temperature, thus allowing measurement of the intra-day difference in temperature. This parameter was shown to be significantly correlated with the number of clinic consultations for NAR, indicating that change in temperature, classically known as a subjective aggravating factor, was actually reflected in the number of clinical consultations for acute exacerbations of NAR. Under the weather conditions characterized by a large intra-day difference in temperature, vasodilation or vasoconstriction for body temperature adjustment can take place following a sharp elevation or reduction in the temperature [28], and imbalance of the autonomic nerve system which regulates the vasomotor tone is anticipated to induce symptoms of rhinitis such as nasal discharge, nasal congestion, and sneezing via increasing the vascular permeability.

In the present study, the atmospheric level of OX was the environmental factor that showed the strongest positive correlation with the number of clinic consultations for NAR. OX is a collective term for oxidizing substances contained in the atmosphere and is responsible for photochemical smog. NOx and HC, released from factories, automobiles, etc., undergo denaturing through “photochemical reactions” when exposed to ultraviolet rays from sunlight, resulting in secondary formation of oxidizing substances, including ozone (O_3_, a primary component), aldehyde (R-CHO), peroxyacetyl nitrite (PAN = R-CO_3_NO_2_), etc. Usually, these oxidizing substances in atmosphere, except NO_2_, are referred to under the collective term “photochemical oxidant (OX)”. Due to the aforementioned mechanism for formation, the OX level is high during the daytime from spring to summer (seasons with strong sunlight), unlike the tendency for the other air pollutants [29]. OX is more likely to be formed during the daytime on fine summer days with weak wind and strong ultraviolet rays. Therefore, OX is considered to serve as an NAR aggravating factor independent of the maximum wind speed which was also found to show a positive correlation with the number of clinical consultations for NAR. Among the meteorological factors, the two factors showing the weak but significant correlation with the number of clinic consultations for NAR (total global solar radiation and duration of daylight) were suggested to probably increase the number of clinic consultations for NAR through accelerated OX formation by ultraviolet rays from sunlight. OX has a strong irritant activity on ocular conjunctiva, nasal mucosa, and upper airways such as bronchi, and an environmental criterion range of OX (1-h level not exceeding 0.06 ppm) has been set by the Ministry of the Environment in Japan. In Japan, there is a system under which the prefectural governor issues a photochemical smog alert pursuant to the Air Pollution Control Law if the 1-h photochemical oxidant level reaches 0.12 ppm or higher (criteria for issuance of the “photochemical smog alert”) and is expected to remain elevated, urging inhabitants to take precautions and requesting large-scale factories/workplaces to reduce the release of air pollutants. According to the environmental data analyzed in the present study, the maximum OX level during the study period was 0.053 ppm, remaining below the environmental criterion range throughout the year, but even exposure to such low levels of OX was found to be correlated with the number of clinic consultations for NAR patients, suggesting that exposure to OX can affect the appearance of rhinitis symptoms in NAR patients even when its level is below the environmental criterion range. The levels of other air pollutants such as nitrogen dioxide (NO_2_) are high in the winter (November through January), during which the atmosphere tends to be stable and weather conditions tend to reduce the diffusion of air pollutants in atmosphere; however, considering that the atmospheric levels of NO, NO_2_, and NOX were negatively correlated with the number of clinical consultations for NAR, it seems probable that pooling of these substances at high concentration levels without assuming the form of secondary product OX does not aggravate NAR and reduces the number of clinical consultations for NAR.

Among the air pollutants other than OX, the atmospheric CO level was significantly correlated with the number of clinic consultations for NAR. Because CO is not directly involved in the formation of OX, it may serve as an independent aggravating factor. This association with atmospheric CO is of interest: CO is abundantly contained in tobacco, which is well known as a classical NAR aggravating factor [9], and poses high stress to the cardiovascular system. However, it has been reported that acute symptoms are unlikely to result from exposure to CO at the level contained in tobacco smoke or at the mean level recorded in the present study (0.23 ± 0.06 ppm), which was much lower than the environmental criterion range for air pollution control in Japan (daily average of 1-h level not exceeding 10 ppm and 8-h average not exceeding 20 ppm) [30, 31]. Despite these facts, the present study revealed a significant correlation between the number of clinic consultations for NAR and the atmospheric CO level. Recently, autonomic imbalance arising from inhalation of tobacco smoke has been reported [10, 32], but its exact mechanism is unknown. Further investigation is desirable to clarify the mechanism of aggravation of NAR following exposure to CO.

Variables for which significant correlation coefficients were also obtained in the multiple regression analysis (*F* = 17.327, *p* < 0.001, *R*^2^ = 0.546) were the mean vapor pressure (coefficient of determination 27.3 %), mean cloud cover (7.5 %), sulfur dioxide (5.7 %), and nitric oxide (9.0 %). These results suggest that the mean vapor pressure, which showed a negative correlation with the number of clinical consultations for NAR, is the factor having the greatest influence on the number of clinical consultations for NAR, indicating that it is a factor aggravating NAR throughout the year, and that drying of the nasal mucosa due to reduction in the ambient vapor pressure may also affect the condition of NAR patients throughout the year. This suggestion does not contradict the clinically common phenomenon that the subjective symptoms of NAR patients can be alleviated by humidifying measures, such as wearing of a mask.

### Discussion of seasonal elements

**Fig. 4:**
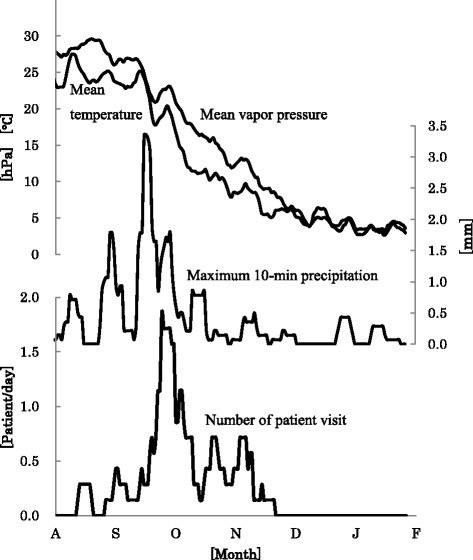
Relationships with the environmental factors in NAR patients who visited the clinic only during the autumn-winter period. The major factors affecting the number of NAR patients were the mean vapor pressure (coefficient of determination 58.4 %), mean temperature (44.4 %), and maximum 10-min precipitation (12.0 %)

In the analysis of the relationships with the environmental factors in NAR patients who visited the clinic only during the autumn-winter period, the major factors affecting the number of NAR patients were the mean vapor pressure (coefficient of determination 58.4 %), mean temperature (44.4 %), and maximum 10-min precipitation (12.0 %) (Fig. 4). This result suggests that the number of clinical consultations tends to increase on warm and high-moisture days with heavy rain. Sudden changes in the vapor pressure during the autumn-winter period (dry and low-temperature condition usually prevailing stably) can stimulate the nasal mucosa. In addition, elevation in vapor pressure and temperature during the autumn-winter period (sweating unlikely to occur) can hamper body temperature control through insensible water loss, resulting in a reduction of the body temperature, dilatation of the peripheral blood vessels (to maintain a normal condition), and fall of blood pressure. The accompanying excessive sympathetic nerve tension can induce symptoms of rhinitis mediated by rebound parasympathetic nerve tensioning, etc., although the details still need to be clarified on.

**Fig. 5:**
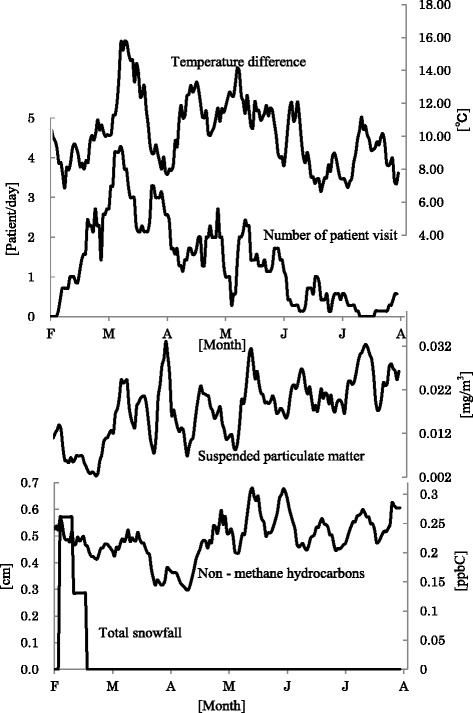
Relationship with environmental factors in NAR patients visiting the clinic only during the spring-summer period. The major parameters with significant Spearman’s correlation coefficients and significant correlation coefficients also in the multiple regression analysis (*F* = 33.053, *p* < 0.001, *R* ^2^ = 0.836) were the temperature difference (coefficient of determination 13.3 %), non-methane hydrocarbons (7.9 %), suspended particulate matter (6.4 %), and total snowfall (4.8 %)

In the analysis of the relationships with the environmental factors in NAR patients who visited the clinic only during the spring-summer period, the major parameters with significant Spearman’s correlation coefficients and significant correlation coefficients also in the multiple regression analysis (*F* = 33.053, *p* < 0.001, *R*^2^ = 0.836) were the temperature difference (coefficient of determination 13.3 %), non-methane hydrocarbons (7.9 %), suspended particulate matter (6.4 %), and total snowfall (4.8 %) (Fig. 5). In the warm and moist climate zone where Japan is located, the circadian variance of the temperature is large (low temperature in the mornings and evenings and high temperature during the daytime) in the winter to spring, with the circadian variance often exceeding 10 °C. These features of climate during the spring-summer period can stress the autonomic nervous system designed to maintain homeostasis. Furthermore, in the spring, Asian dust reaches Japan from the Eurasia Continent, possibly stimulating the nasal mucosa through increase in the suspended particulate matter. As evidence supporting this view, the present study revealed a decrease in the number of clinical consultations for NAR in our district in the early spring when snowfall was frequently seen.

## Conclusions

The atmospheric levels of OX and CO (among the air pollutants) and sudden changes in the weather involving strong winds (among the meteorological factors) showed a significant positive correlation with the number of clinic consultations for NAR in the present study. Major parameters with significant Spearman’s correlation coefficients and significant correlation coefficients also in the multiple regression analysis were the mean vapor pressure (coefficient of determination 27.3 %) throughout the year, mean vapor pressure (58.4 %), mean temperature (44.4 %), maximum 10-min precipitation (12.0 %) only during the autumn-winter period, and temperature difference (13.3 %) only during the spring-summer period. In conclusion, the results of the present study suggest that the mean vapor pressure is the most important environmental factor associated with acute exacerbation of NAR. The mechanisms underlying the association between these aggravating factors and NAR exacerbations remain to be clarified. Further studies are desirable to examine whether or not acute exacerbations of NAR can be prevented by avoiding going outdoors on days with unfavorable meteorological conditions or air pollutant levels and adjusting the environments making use of air conditioners or air cleaners.

## References

[1] Fokkens WJ (2002). Thoughts on the pathophysiology of nonallergic rhinitis. Curr Allergy Asthma Rep..

[2] Wallace DV, Dykewicz MS, Bernstein DI (2008). The diagnosis and management of rhinitis: an updated practice parameter. J Allergy Clin Immunol..

[3] Bachert C (2004). Persistent rhinitis—allergic or nonallergic?. Allergy..

[4] Bousquet J, Fokkens W, Burney P (2008). Important research questions in allergy and related diseases: nonallergic rhinitis: a GA2LEN paper. Allergy..

[5] Newhall KK, McGrath KG (2004). Persistent rhinitis—allergic or nonallergic?. Allergy Asthma Proc..

[6] Nassef M, Shaoiro G, Casale TB (2006). Respiratory and Allergic Disease Foundation. Identifying and managing rhinitis and its subtypes: allergic and nonallergic components—a consensus report and materials from the Respiratory and Allergic Disease Foundation. Curr Med Res Opin.

[7] Kaliner MA, Baraniuk JN, Benninger M (2009). Consensus definition of nonallergic rhinopathy, previously referred to as vasomotor rhinitis, nonallergic rhinitis, and/or idiopathic rhinitis. WAO J..

[8] Smolensky MH, Lemmer B, Reinberg AE (2007). Chronobiology and chronotherapy of allergic rhinitis and bronchial asthma. Advanced Drug Delivery Reviews..

[9] Shusterman D, Murphy MA (2007). Nasal hyperreactivity in allergic and non-allergic rhinitis: a potential risk factor for non-specific building-related illness. Indoor Air..

[10] Skoner DP (2001). Allergic rhinitis: definition, epidemiology, pathophysiology, detection, and diagnosis. J Allergy Clin Immunol..

[11] Pattanaik D, Lieberman P (2010). Vasomotor rhinitis. Curr Allergy Asthma Rep..

[12] Bernstein JA (2009). Characteristics of nonallergic vasomotor rhinitis. World Allergy Organ J..

[13] Ma Y, Tan G, Zhao Z (2014). Therapeutic effectiveness of endoscopic vidian neurectomy for the treatment of vasomotor rhinitis. Acta Otolaryngol..

[14] Takeno S, Noda N, Hirakawa K (2012). Measurements of nasal fractional exhaled nitric oxide with a hand-held device in patients with allergic rhinitis: relation to cedar pollen dispersion and laser surgery. Allergol Int..

[15] Ciprandi G (2004). Treatment of nonallergic perennial rhinitis. Allergy..

[16] Garay R (2004). Mechanisms of vasomotor rhinitis. Allergy..

[17] Tan G, Ma Y, Li H, Li W, Wang J (2012). Long-term results of bilateral endoscopic vidian neurectomy in the management of moderate to severe persistent allergic rhinitis. Arch Otolaryngol Head Neck Surg..

[18] Scarupa MD, Kaliner MA (2009). Nonallergic rhinitis, with a focus on vasomotor rhinitis clinical importance, differential diagnosis, and effective treatment recommendations. World Allergy Organ J..

[19] Brandt D, Bernstein JA (2006). Questionnaire evaluation and risk factor identification for nonallergic vasomotor rhinitis. Ann Allergy Asthma Immunol..

[20] Segara C, Fauroux B, Just J, PAscual L, Grimfeld A, Neukirch F (1998). Short-term effect of winter air pollution on respiratory health of asthmatic children in Paris. Eur Respir J..

[21] Sydbom A, Blomberg A, Parnia S (2001). Health effects of diesel exhaust emissions. Eur Respir J..

[22] Samet JM, Dominici F, Curriero FC (2000). Fine particulate air pollution and mortality in 20 U.S.cities, 1987–1994. N Engl J Med.

[23] Osunsanya T, Prescott G, Seaton A (2001). Acute respiratory effects of particles: mass or number?. Occuo Eviron Med..

[24] Hoshino T, Hoshino A, Matsubara N (2011). Relationship between the number of outpatient visits for hypotension in the springtime in Japan, extracted from clinical electronic records, and global solar radiation levels. J Int Med Res..

[25] Zhang Y, Peng L, Kan H, Xu J, Chen R, Liu Y (2014). Effects of meteorological factors on daily hospital admissions for asthma in adults: a time-series analysis. PLoS One..

[26] Hyrkäs H, Jaakkola MS, Ikäheimo TM, Hugg TT, Jaakkola JJ (2014). Asthma and allergic rhinitis increase respiratory symptoms in cold weather among young adults. Respir Med..

[27] Villeneuve PJ, Leech J, Bourque D (2005). Frequency of emergency room visits for childhood asthma in Ottawa, Canada: the role of weather. Int J Biometeorol..

[28] Sanchez-Gonzalez MA, Figueroa A (2013). Cold exposure attenuates post exercise cardiovagal reactivation and sympathetic withdrawal. Auton Neurosci..

[29] Li P, Xin J, Bai X, Wang Y, Wang S, Liu S (2013). Observational studies and a statistical early warning of surface ozone pollution in Tangshan, the largest heavy industry city of North China. Int J Environ Res Public Health..

[30] Verrier A, Daoudi J, Gourier-Frry C (2010). Salines carbon monoxide poisoning surveillance: a French Environmental & Health surveillance system integrated in preventive policies G. Inj Prev..

[31] Rapley C (2012). The health impacts of climate change. BMJ..

[32] Middlekauff HR, Park J, Agrawal H, Gornbein JA (2013). Abnormal sympathetic nerve activity in women exposed to cigarette smoke: a potential mechanism to explain increased cardiac risk. Am J Physiol Heart Circ Physiol..

